# Analgesia for the Bayesian Brain: How Predictive Coding Offers Insights Into the Subjectivity of Pain

**DOI:** 10.1007/s11916-023-01122-5

**Published:** 2023-07-08

**Authors:** Friedrich E. Lersch, Fabienne C. S. Frickmann, Richard D. Urman, Gabriel Burgermeister, Kaya Siercks, Markus M. Luedi, Sven Straumann

**Affiliations:** 1 Department of Anaesthesiology and Pain Medicine, Cantonal Hospital of St. Gallen, St. Gallen, Switzerland; 2 Department of Anaesthesiology and Pain Medicine, Inselspital, Bern University Hospital, University of Bern, Freiburgstrasse, 3010 Bern, Switzerland; 3 Department of Anesthesiology, The Ohio State University, Columbus, OH 43210 USA

**Keywords:** Predictive coding, Analgesia, Pain, Anesthesia, Active inference, Bayes’ theorem, Markov blanket

## Abstract

**Purpose of Review:**

In order to better treat pain, we must understand its architecture and pathways. Many modulatory approaches of pain management strategies are only poorly understood. This review aims to provide a theoretical framework of pain perception and modulation in order to assist in clinical understanding and research of analgesia and anesthesia.

**Recent Findings:**

Limitations of traditional models for pain have driven the application of new data analysis models. The Bayesian principle of predictive coding has found increasing application in neuroscientific research, providing a promising theoretical background for the principles of consciousness and perception. It can be applied to the subjective perception of pain.

**Summary:**

Pain perception can be viewed as a continuous hierarchical process of bottom-up sensory inputs colliding with top-down modulations and prior experiences, involving multiple cortical and subcortical hubs of the pain matrix. Predictive coding provides a mathematical model for this interplay.

## Introduction

To effectively treat pain, we must first understand it. How it arises from the integration of nociceptive signals into the nervous system is at the heart of anesthesiologists’ and pain therapists’ work [1••]. One theory of consciousness—“predictive coding” in the model of the Bayesian brain [2, 3, 4••]—lends itself well to discussing the perception of both pain and analgesia. Named after the statistician Bayes, the theory is based on conditional probabilities. It is the aim of this article to discuss recent neuroscientific research within the framework of hierarchical Bayesian inference and to apply the concepts of predictive coding to pain perception. This review aims to help clinicians understand the mechanisms of pain perception and analgesia from a neuroscientific perspective.

The theory of predictive coding (PC) draws on a variety of concepts from biophysics, information theory, and statistics. While this offers novel ways of understanding perception, it may also evoke confusion due to nomenclature unfamiliar to medical clinicians. Hence, we think it is prudent to first outline three fundamental concepts. We will use the nomenclature established here to apply PC to the perception of pain and analgesia in subsequent sections.

**Fig. 1:**
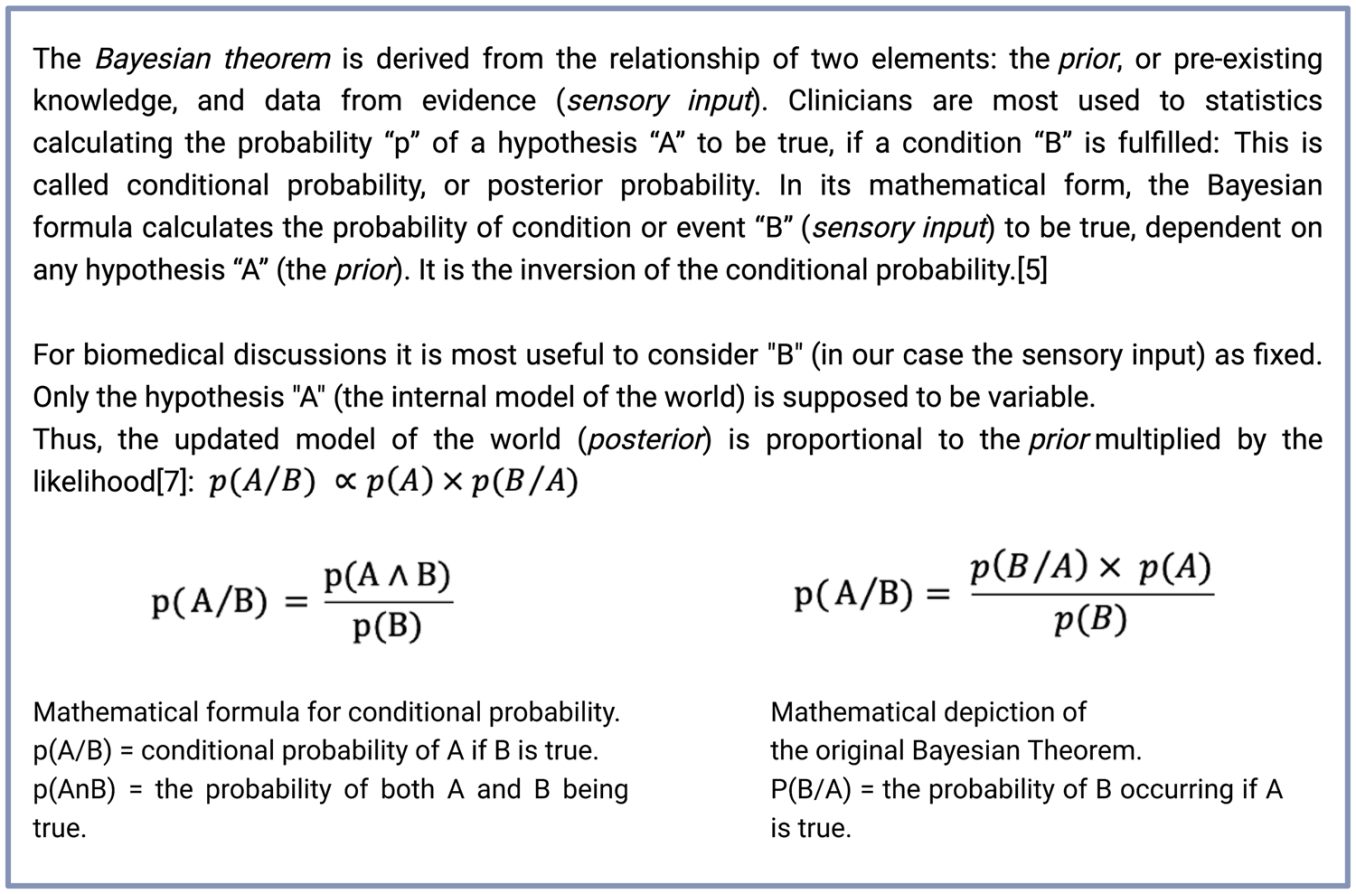
Excursus into the statistical background of the Bayesian theorem

1. *Bayesian* or *active inference* is a form of statistical data analysis in which the probability of a hypothesis is continually updated according to newly arising evidence. The analysis is based on *Bayes’ theorem* (refer to Fig. 1 for a statistical explanation). In neuroscience, active inference can be used to explain perception not as simple input–output processing, but as an *internal model* of the exterior world that is updated if *sensory input* does not correspond to it [5]. The internal model is called the *prior*, the updated model the *posterior*. The general idea that the brain’s main functions rely on this type of active inference is called *predictive coding* or *hierarchical Bayesian inference* [5].
2. *Predictive error* is the difference between a *prior* (i.e., how we expect the world to be) and the actual sensory input. It is a measure of *surprisal*. In the *free energy principle*, as postulated by Friston et al., the term free energy is often used synonymously with predictive error (PE) in this sense [6]. A core concept of the free energy principle is that the brain uses predictive coding to minimize free energy, i.e., to build models of the surrounding world that are accurate enough to predict most of what the organism will encounter. It does so to keep the organism within safe boundaries that support its survival [6, 7•]. A basic example of this is temperature. We avoid touching stoves and being in blizzards because our internal model accurately predicts that the temperatures we would encounter would lie outside of our biological boundaries. Hence, we optimize our survival by minimizing free energy.
3. *Markov blanket* is a statistical term describing a set of nodes in a network that provide all information about the nodes situated inside of the network. In the context of PC, it may be helpful to view the Markov blanket as all structures that separate an internal system from its surroundings [7•]. Markov blankets can be applied to both micro- and macroscales. The cell wall is an intuitive form of a Markov blanket on a microscale since it separates all interior processes of the living cell from its surroundings. In general, internal processes are kept in homeostasis by the organism, while external processes are more apt to be influenced by entropy. Thus, the concept of Markov blankets provides a mathematical model to differentiate between the subject and the world it tries to navigate [8].

## From Nociception to Pain

Increasing numbers of patients seem to embark on a trajectory of chronic postoperative pain [9–11]. The opioid crisis and different problematic aspects of our analgesic practice [12] urge us to reconsider our concept of analgesia. Recent neuroscientific findings shape promising new concepts of the biological machinery underlying consciousness and the mind [6, 13, 14, 15•].

Any organism strives for stable homeostasis to survive. It is not simple nociception that drives our behavior but the *affective valence* we award nociception (i.e., the feeling that arises due to it). This affective valence is constantly created and re-evaluated. Aberrations from homeostasis, such as extreme temperature (exteroceptive), hypoglycemia, or acidosis (interoceptive), induce acute changes in primordial emotions such as hunger, thirst, pain, or pleasure [16, 17].

In the case of pain, data input that facilitates its evaluation and re-evaluation is gained through two principal paths: *nociception* and *modulating factors*. The detection of (potential) tissue damage is the bedrock of pain perception. Yet, only in combination with input from a wealth of modulating neurobiological networks, such as the limbic system, the striatum or the periaqueductal gray do we eventually assign it affective valence [1••, 18–20, 21••].

## A Predictive Coding Model for Subjective Pain

A simplified predictive coding model of perception in general, modified from Chen and Wang [21••], can be expressed as follows (Fig. 3):

$$perception=prior+gain \times predictive\; error$$

The prior internal model we hold for a certain situation is the basis of perception. We then add the predictive error (PE), i.e., sensory input that does not match our prior. Crucially, the PE is modulated by a factor *gain* comprising any circumstances that make us alter how we let surprisal affect our expectations. This relationship will become clearer when applied to nociception and pain [22, 23].

$$subjective\; pain=prior+gain \times negative\; affective\; valence$$

When applying PC to pain perception, negative affective valence can be viewed as the principal PE. It is what urges the re-evaluation of a prior model of homeostatic tranquility. If the PE is low, we perceive the situation just as we would have expected to perceive it prior to nociception [23].

Building on this model, we propose negative affective valence can be further partitioned into bottom-up nociception and top-down modulation. Their ratio denotes *precision*, a factor determining whether the input will stay below or surpass a threshold value of pain perception [21••]:

$$subjective\; pain=prior+gain \times \left(\frac{bottom-up\; nociception}{top-down\; modulation}\right)$$

It is important to note the difference between gain and top-down modulation in our model.

*Gain* defines how much the predictive error influences our perception through factors such as attention and motivation. It does not alter the affective valence we assign to the PE, but how much we let it in turn affect our prior. A good example of this is regional anesthesia (RA). Any nociceptive stimulus we still perceive will hold the same negative affective valence as before the start of anesthesia, but the intensity of that feeling is dramatically reduced, preferably to a point where the gain equals 0. Sedation or opioid analgesia can also generally be assumed to reduce gain for an incoming nociceptive stimulus.

Top-down modulation, on the other hand, changes the affective valence we assign to a bottom-up stimulus. An example of this is motivation during physical exercise. Nociceptive signals arising from strained muscles and lungs are no longer interpreted as something negative; hence, we do not perceive them as subjective pain. Ketamine can also alter top-down modulation through psychological dissociation and an increased pain-threshold, especially when administered with anxiety-reducing drugs or positive suggestions and pleasant music.

In keeping with the tenets of active inference, it is important to note how gain and modulation influence not only the current perception of pain but also the alteration of the prior to form a new posterior. If we provide analgesia through any of those two pathways, we will not only decrease a patient’s momentary discomfort but also shape their expectation toward upcoming similar situations.

To paint a clearer picture, let us apply this model to orthopedic osteosynthesis under RA. The nociceptive input may be completely suppressed through a peripheral nerve block (gain). But if the patient has been in pain, is very anxious, or stressed (modulation), simple touch or proprioception (bottom-up) induces negative emotional valence and may trigger subjective pain. This not only causes discomfort for patients but also alters their *prior* to such extent that their perceived pain will only exacerbate in a similar situation in the future because their posterior will already hold a high amount of negative affective valence.

On the other hand, anxiolytic techniques such as hypnosis, meditation, or music may reduce the precision by increasing the influence of top-down modulation [24, 25]. In this case, no subjective pain will arise, since the patient’s *prior* (“being without pain”) remains unchanged.

**Fig. 2:**
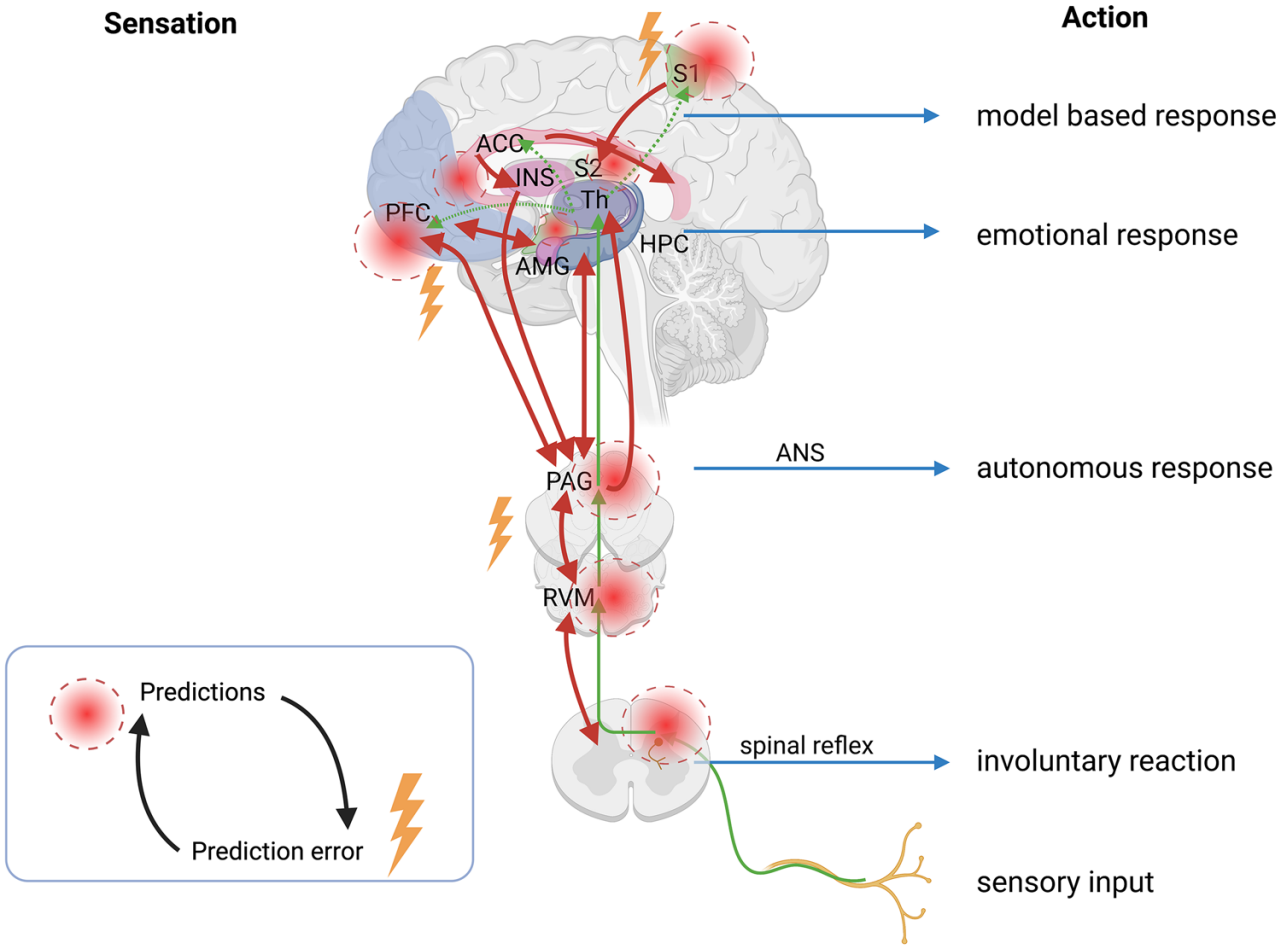
The hierarchical principle of predictive coding applied to the pain pathway. At each level, predictions about the world are formed and sent to the level below (red arrows), where they are compared to the incoming information (green arrows). A prediction error is calculated and sent back to the level above, where the *prior* is updated according to the new information. Actions of varying voluntary control can arise at each level of the hierarchy, from primitive reflexes to reflected reactions. (ANS, autonomous nervous system; RF, reticular formation; PAG, periaqueductal gray; RVM, rostral ventromedial medulla; INS, insular cortex; ACC, anterior cingulate cortex; PFC, prefrontal cortex; S1, primary somatosensory cortex; S2, secondary somatosensory cortex; AMG, amygdala; Th, thalamus; HPC, hippocampus)

Here it is useful to stress the “hierarchical” aspect of PC. As the incoming nociceptive perception climbs the rungs of the bottom-up integrational pathway, its significance can be altered on each level, from dermal receptors to the dorsal horn via brainstem, subcortical networks to the limbic system, the thalamus, and finally neocortex (Fig. 2). This affords ample possibilities to halt the progression of nociception in a multi-modal strategy.

To name some examples, experienced meditators do not show pain reactions to thermal stimuli that cause significant pain in non-meditators [26]. Hypnotic suggestions reduce subjective pain in standardized pain stimuli along with causing alterations in fMRI patterns of the cingulate gyrus [27], parietal operculum, or the insula [28]. Listening to pleasant music will deflate the surprisal of a stimulus and though that its potential for pain and suffering [29, 30•].

## Applying Our Model to Pain Therapy

To apply our simplified PC formula to analgesia, one more crucial factor needs to be considered: time. Here the application of a Markov blanket comes into the picture. The perception of pain takes place within a confined system, which is shielded from its surroundings by a Markov blanket [20]. Notably, the Markov blanket is not equal to the anatomical separation of the brain from the outside world but comprises all systems that provide predictive error to the equation [8]. Interoceptive sensations, such as expectations and fear, are as much part of the Markov blanket as exteroception, such as nociception or visual input.

**Fig. 3:**
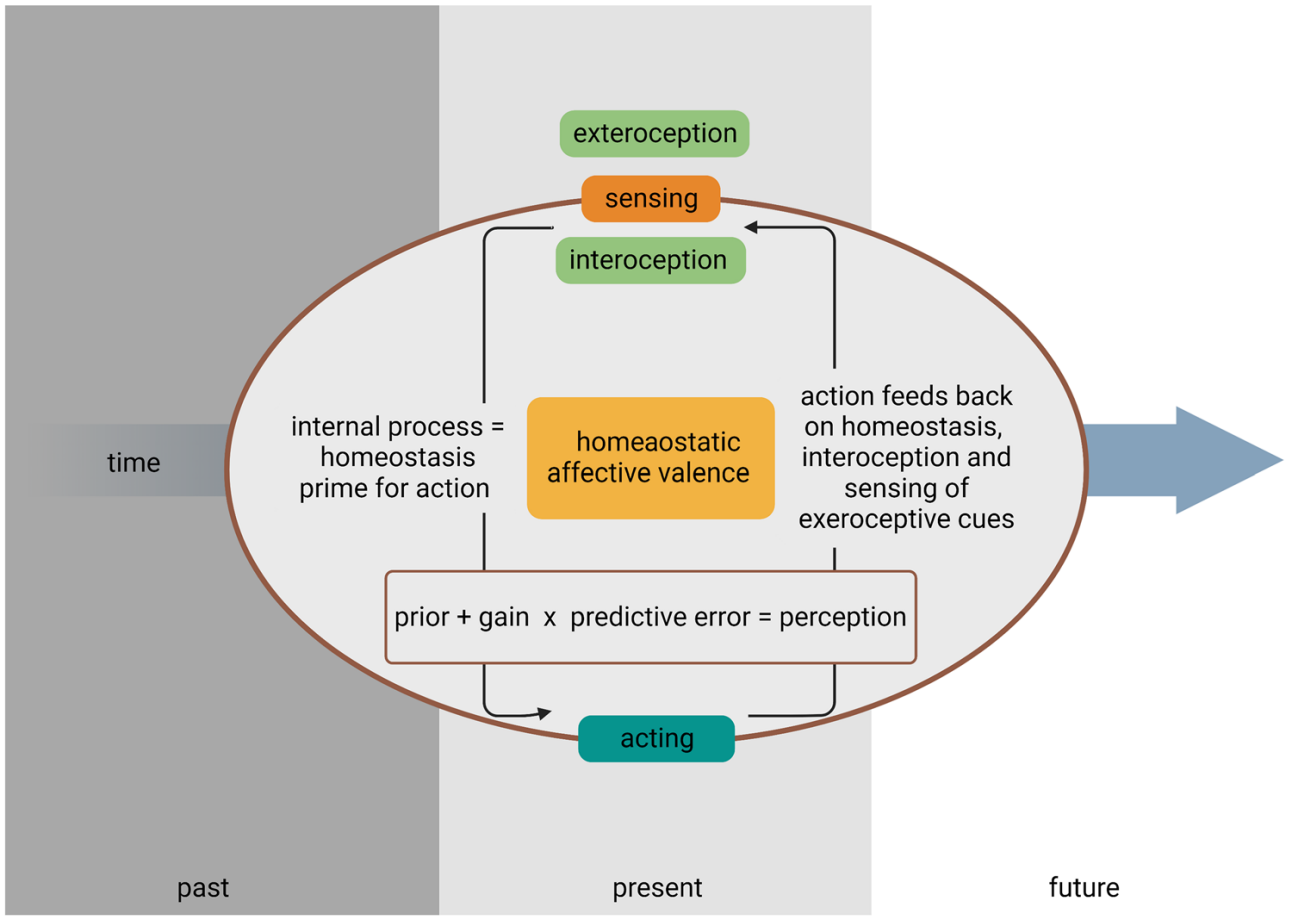
The Markov blanket of perception

The Markov blanket persists over time and draws on sensory input (*sensing*) to formulate an adequate response (*acting*). It furthermore updates its posterior, so it can minimize its free energy (i.e., surprisal) in the future (Fig. 3) [31].

**Fig. 4:**
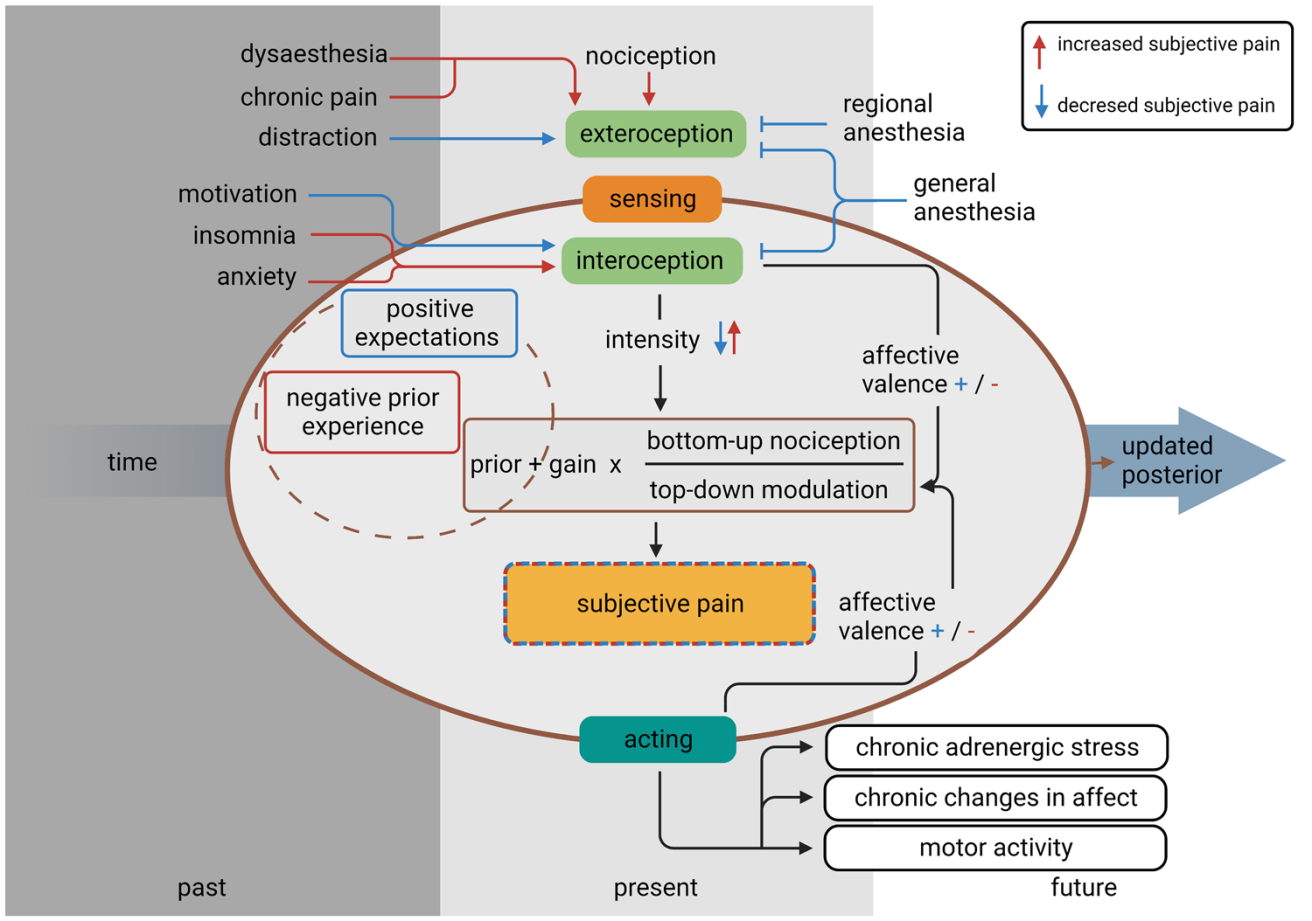
The Markov blanket of pain perception

Now the variables in our model can be filled with concrete examples of pain perception and analgesia (Fig. 4). The bottom-up nociception is readily understood. It corresponds to any sensory input not already contained in the prior. In our case, this represents unexpected nociceptive signals. During a surgical procedure, such signals will invariably arise, and it is in our hands to modulate them to alter the impact on subjective pain.

The prior corresponds to how a patient expects to feel. We are not able to instantly change a patient’s prior. However, any sufficiently large PE will alter their posterior. It is in our hands to help the patient experience positive affective valence associated with any PE. If we keep showing the brain that its current prior overestimates the level of subjective pain, the posterior will gradually decrease.

After having introduced some modulating factors, such as motivation or ketamine-analgesia as top-down modulating factors and RA and anxiety as modulators of gain, other important modulators of the gain function are the attention to or distraction from pain [21••], neurochemical changes in the affected tissue such as inflammation [32] and chronic pain, which can trigger neurogenic inflammation through, among other mechanisms, the release of substance P, increasing the gain [33]. The list of factors that can influence the gain and top-down modulation is too vast to be listed in this article. However, our simplified model can be used as scaffolding to describe how any analgesic technique or mental state influences subjective pain. The importance of expectation management and anxiolysis at the outset of any perioperative situation cannot be stressed enough [34].

## Applying Our Model to General Anesthesia

As demonstrated above, different variables in pain perception can be modulated selectively while a patient is awake or only lightly sedated.

As the emotional valence lies at the heart of this consciousness theory, strategies that target stress responses, anxiety, and pain evaluation need to be incorporated as filters in our model, even in the setting of general anesthesia (GA). As visualized by the Markov blanket, non-pharmacological analgesic strategies, as well as pharmacological sedation and GA all modulate the sensing and the acting state of the blanket. It can be argued that the homeostatic affective valence a patient holds is gradually reduced through GA, but not entirely erased. This idea has been previously explored in concepts of core consciousness under GA [35]. We know that different degrees of unconsciousness can still allow for pain perception, even while the individual is externally unresponsive to painful stimuli [36].

Whether or not patients retain the ability for predictive coding under GA is inherently difficult to study. However, changes in subjective perception often go hand in hand with distinct electroencephalographical (EEG) patterns. These may provide insight into the perception of a patient under GA. Slow waves such as those in the delta (0.5–4 Hz) and theta (4–7 Hz) range, as well as alpha oscillations (8–13 Hz), suggest sufficient anesthesia and analgesia. Emerging beta waves (14–29 Hz), as described in analgetic hypnotic states [37] or analgetic musical chills [38], could be a signal of arousal under anesthesia. When ketamine is co-administered with propofol and dexmedetomidine during multimodal GA, it often alters the EEG-spectrum from the typical propofol alpha-delta-pattern to one reduced in delta-power but with increasing power in the low beta-range. We hypothesize that this ketamine-induced brain activity may be the hallmark of preserved PC in the brain during GA.

The neurobiological templates on which PC unfolds seem to be canonical cortical networks. Lower cortical layers (i.e., layer V) are presumed to code existing prior models by slow oscillations [39]. Superficial layers, oscillating in the gamma and beta ranges, may code prediction errors. This raises intriguing questions. Do these different states of cortical oscillation states have any bearing on active inference during general anesthesia? [40]. Can they transport previous factors of top-down modulations and gain, such as pre-operative anxiety, all the way to the recovery room? Or is all the effort of pre-operative expectation management, anxiolysis, and non-pharmacological analgesia drowned in layer V delta waves that are the end state of most anesthetic agents at sufficiently high doses [41].

The analysis of different activation states of the thalamocortical level and the brain stem suggests that even core consciousness is annihilated during long spans of burst suppression. While this makes it highly unlikely for the brain to uphold a capacity for PC in such states, the same may not be true in more superficial levels of GA, which are often sufficient for most surgical procedures. It may be plausible to inquire into the role of dreams and retained—albeit heavily altered—consciousness under GA through the concept of PC. Obviously, such states of consciousness are unlikely connected to the exterior world [42, 43], but they may still be engaged in a therapeutic manner through retention of gain and modulating factors, implemented before putting the brain under general anesthesia [44, 45•]. Raw EEG patterns during maintenance and emergence determine important postoperative endpoints such as delirium [46] and pain [47]. This could be interpreted as a sign of residual active inference capacities during spindle (alpha-) rich anesthesia [48].

This proposition is strengthened by the strong correlation between preoperative anxiety and postoperative pain [49, 50]. Nelson et al. recently demonstrated the inverse effect: expectation management and preoperative anxiolysis positively influence postoperative pain [51].

Drawing the line where PC abates completely during anesthetic unconsciousness is highly hypothetical. Alongside future studies during GA, analogies to pain experiences in disorders of consciousness, such as vegetative or minimally conscious states, may improve our understanding both in critical care and anesthesia practice [52]. Understanding that the regulation of neuronal networks and coding may be partially independent from wakefulness may aid our understanding why patients can be unresponsive but still in pain.

## Conclusion

Although predictive coding and the underlying free energy principle may not end up being the universal theory of the brain [6], it offers a conceptual scaffolding for the clinical practice of anesthesiologists and pain therapists. Recent research suggests we may further our understanding of pain management by analyzing subjective pain through the lens of predictive coding. The brain may retain the function of active inference even during deep sedation and general anesthesia, which may explain why preoperative analgetic techniques such as anxiolysis and expectation management can help curb postoperative pain.

Our simplified predictive coding model is based on our literature research and our personal insights and considerations. We are convinced it is useful to both clinicians and researchers by offering explanations and descriptions of how subjective pain arises through a wealth of modulating factors. We think mathematical models, even when simplified, are efficient and precise tools for communicating and researching pain and analgesia from a neuroscientific perspective.
